# From Learning to Memory: A Comparison Between Verbal and Non-verbal Skills in 22q11.2 Deletion Syndrome

**DOI:** 10.3389/fpsyt.2021.597681

**Published:** 2021-06-16

**Authors:** Johanna Maeder, Mathilde Bostelmann, Maude Schneider, Karin Bortolin, Matthias Kliegel, Stephan Eliez

**Affiliations:** ^1^Developmental Imaging and Psychopathology Lab, Department of Psychiatry, University of Geneva School of Medicine, Geneva, Switzerland; ^2^Laboratory of Brain and Cognitive Development, Institute of Psychology, University of Lausanne, Lausanne, Switzerland; ^3^Clinical Psychology Unit for Intellectual and Developmental Disabilities, Faculty of Psychology and Educational Sciences, University of Geneva, Geneva, Switzerland; ^4^Center for Contextual Psychiatry, Department of Neurosciences, KU Leuven, Leuven, Belgium; ^5^Medical Image Processing Laboratory, Institute of Bioengineering, École Polytechnique Fédérale de Lausanne, Lausanne, Switzerland; ^6^Center for the Interdisciplinary Study of Gerontology and Vulnerability, and Swiss National Center of Competences in Research LIVES–Overcoming Vulnerability: Life Course Perspectives, Geneva, Switzerland; ^7^Cognitive Aging Lab, Department of Psychology, University of Geneva, Geneva, Switzerland; ^8^Department of Genetic Medicine and Development, University of Geneva, Geneva, Switzerland

**Keywords:** 22q11.2 deletion syndrome, memory, learning, retention, recognition, genetic condition

## Abstract

**Background:** Previous studies on possible memory deficits in 22q11DS often focused on quantifying the information memorized, whereas learning processes have been mostly overlooked. Furthermore, methodological differences in task design have made verbal and non-verbal comparison challenging and mixed results have been observed depending on chosen stimuli.

**Method:** 135 participants (78 with 22q11DS) completed a multi-trial memory task modeled after the Rey Auditory Verbal Learning Task, comparing verbal and non-verbal learning as well as retention over time. Performance in the 22q11DS group were compared to controls and learning curves were analyzed.

**Results:** In 22q11DS, slower acquisition of non-verbal material and higher rates of errors in both verbal and non-verbal tasks was observed. After 30 min, free recall performance, when corrected for initial learning rate, was similar between 22q11DS and controls. Conversely, recognition performance was overall weaker for 22q11DS in both modalities (verbal and non-verbal).

**Conclusion:** This study examined how information is acquired, retained in memory over time and how different recall modalities (free recall vs. recognition) could yield different performances. Clinical implications of the findings are discussed.

## Introduction

Cognitive deficits and learning disabilities are hallmarks of chromosome 22q11.2 deletion syndrome (22q11DS). Needs for educational assistance and specialized education are frequent and tend to increase with age (1). To improve management and care for these patients, a comprehensive picture of their neuropsychological profile is necessary. By studying how information is acquired and retained over time, specific recommendation and rehabilitation tools can be tailored to fit patients' needs.

Studies exploring memory functioning in 22q11DS have consistently reported impaired non-verbal memory performance, whereas verbal memory appeared to be less affected, or even preserved compared to general intellectual functioning (i.e., IQ scores) (2–4). A wide range of tasks have been used to assess memory functioning in this population, sometimes merging performance of several tasks into a single verbal or non-verbal index, or even a general memory score [e.g., Children Memory Scale or Wechsler Memory Scale; (5–7)]. This approach may be problematic as these tasks recruit a variety of different cognitive processes and thus may blur differential effects (e.g., free recall vs. recognition, associative learning vs. implicit learning, isolated vs. complex stimuli). Moreover, directly comparing verbal and non-verbal performance has been a challenge in 22q11DS and should require parallel task designs to draw conclusions.

Memory is a dynamic process, requiring different successive steps: acquiring (encoding), storing (consolidation) and retrieving information (8, 9). Memory impairment can occur from failure at any step but only encoding and retrieval are directly measurable with standard cognitive tasks. In 22q11DS, only few studies have investigated the different components, dissociating encoding from retrieval. Indeed, most studies only considered measures of the quantity of information memorized whereas how information is acquired (learning) has been overlooked. In the verbal modality, the approach mostly used is a wordlist, which provides different quantitative scores of how many items were learned and retained immediately or after a delay in time. Debbané et al. (10) showed that encoding of words during a directed forgetting paradigm is intact in adolescents and young adults with 22q11DS. As part of a larger examination of the neurocognitive profile of 22q11DS, two independent studies in children and adolescents showed (using wordlists) significantly lower performance on learning, immediate recall and delayed recall compared to typically developing individuals (11, 12). However, standardized scores did not show any deficits, except for the recognition score (examined only in 12) which was impaired (z = −1.80). Furthermore, the magnitude of the difficulties seemed consistent with the level expected from IQ differences. In adults, Fiksinski et al. (35) found significant impairment in the sum of words recalled across five trials (z = −1.94), indicating poorer acquisition of verbal information in 22q11DS. Additionally, they showed weaker performance on recall immediately after an interfering list of words (z = −1.53) and preserved immediate recognition performance (z = −0.19). Unfortunately, performance after a delay in time was not examined.

In the visual or non-verbal modality, as reviewed above, tasks are very heterogenous, ranging from dot localization to face recognition, landscapes/scenes or complex figures memorisation. Furthermore, previous results have shown that the degree of impairment in non-verbal memory tasks varies depending on the type of stimuli, with greater deficits for more abstract or complex material (e.g., faces or landscape vs. dot localization) (13). A study using eye-tracking showed atypical exploration strategies (more time spent in the center of the image, less on the peripheral details), leading to sub-optimal encoding and subsequently poorer memory performance (14). Only few studies investigated encoding in 22q11DS using drawings. Antshel et al. (48) observed lower performance for children and adolescents in a complex drawing task compared to typically developing controls. Similarly, Fiksinski et al. (35) demonstrated lower performance in immediate and delayed reproduction of five drawings in adults. Bostelmann et al. (14) highlighted impaired encoding on a drawing reproduction task, related to an abnormal pattern of visual exploration measured with the eye-tracking technique.

To our knowledge, so far only one study has examined verbal and non-verbal learning using parallel task design (15). This study shed light on the dynamic of information acquisition over time by examining learning curves. The authors observed a progression in the acquisition of new information with repetition in both modalities but demonstrated slower acquisition in the non-verbal task. However, the sample of the study was limited, and patterns of errors were only analyzed in the non-verbal task.

In light of the literature reviewed here, the current study is the first to investigate information acquisition by analyzing both pace of acquisition as well as errors during acquisition. Additionally, the design gives the possibility to examine retention over time with multiple complementary measures (free recall and recognition). Furthermore, use of a parallel task design between modalities (verbal vs. non-verbal) provides the opportunity to directly compare performances. Finally, this was done in a large sample of patients with 22q11DS compared to age matched controls.

The first aim was to investigate information acquisition through pace (learning curves) and error rates. According to previous research (15), we hypothesized that the non-verbal learning curve would increase after each trial but be globally weaker (lower from the start and less evolution with repetition) in the 22q11DS group compared to controls. We also expected more errors in 22q11DS. Following results from Debbané et al. (10), we predicted similar performance on verbal learning between both groups, with the same amount of information acquired at the same pace. Due to poorer verbal working memory [e.g., (16–18)], we expected higher rates of errors in 22q11DS during learning.

The second aim was to examine retention of information over time using two types of complementary measures: free recall and recognition. Again, patterns of errors were analyzed. For retention, and according to previous results (4, 13, 15), we excepted preserved verbal retention (comparable to controls), with similar pattern of errors, but impaired non-verbal retention performance in the 22q11DS group, with a significantly higher number of errors.

## Methods

### Participants

One hundred and thirty-five participants, aged 8–25 years old, were recruited from the longitudinal cohort on 22q11DS [e.g., (19, 20)]. Seventy-eight (57.78%) were diagnosed with 22q11DS. All participants completed the learning and memory assessment once, in a cross-sectional design. Due to technical issues (errors in the task instructions or answers not discriminable due to poor articulation), five results (3.70%) had to be excluded from the verbal task and four results (2.96%) from the non-verbal task (see Table 1). This dataset overlaps a previous study focusing on memory consolidation, where learning was briefly discussed only in verbal modality [see (21)]. Compared to the previous sample, 54 (40%) additional new participants (33 with 22q11DS) completed the learning and memory task.

**Table 1:** Participant characteristics.

			**Diagnostic group**		**Comparison**		
			**22q11.2DS**	**Controls**	**ANOVA**	**Pearson's Chi-square**	***P*-value**
*N*			78	57			
Gender [male (%)]			41 (52.56%)	26 (45.61%)		0.636	0.425
Age [mean (SD)]			15.98 (5.19)	14.70 (5.12)	2.025		0.157
Full Scale IQ [mean (SD)]			72.65 (13.22)	114.51 (13.30)	332.12		**<0.001**
Psychiatric diagnosis (%)	Total		45 (57.69%)				
	Categories	Psychosis	2 (2.56%)				
		Attention deficit disorder	24 (30.77%)				
		Simple phobia	21 (26.92%)				
		Social phobia	3 (3.85%)				
		Generalized anxiety disorder	13 (16.67%)				
		Separation anxiety disorder	3 (3.85%)				
		Major depressive episode	4 (5.13%)				
		Obsessive-compulsive disorder	2 (2.56%)				
		Oppositional defiant disorder	3 (3.85%)				
Medication (%)	Total		29 (37.18%)				
	Categories	Methylphenidate	13 (16.67%)				
		Antidepressants	15 (19.23%)				
		Antipsychotics	11 (14.10%)				
		Antiepileptic	3 (3.85%)				
		Anxiolytics	2 (2.56%)				
Verbal task complete (%)			74 (94.87%)	56 (98.24%)			
Non-verbal task complete (%)			77 (98.71%)	54 (94.73%)			
Both tasks complete (%)			73 (93.59%)	53 (92.98%)			

*Significant values at the 0.05 level are displayed in **bold***.

The presence of the deletion was confirmed using quantitative fluorescent polymerase chain reaction (QF-PCR). The typically developing control group was composed of siblings of the affected participants (89.47%) and community controls [see (22)]. Participants from both groups did not differ in terms of age or gender distribution (see Table 1). All participants were recruited through advertisement in patient association newsletters and word-of-mouth. Written informed consent, based on protocols approved by the Ethical Committee of the Canton of Geneva (CCER, Switzerland) was obtained for all participants and their parents (if participant younger than 18 years). For 22q11DS individuals, inclusion was based on a screening interview with SE confirming sufficient verbal comprehension skills to follow task instructions. At time of testing, 45 (57.69%) of 22q11DS patients had at least one psychiatric diagnosis and 29 (37.18%) were taking medication (for details see Table 1). Typically developing controls were screened for psychiatric illnesses, learning disabilities and psychotropic medication prior to inclusion in the study.

### Materials

#### Learning and Memory: Task Specifics and Administration

**Figure 1:**
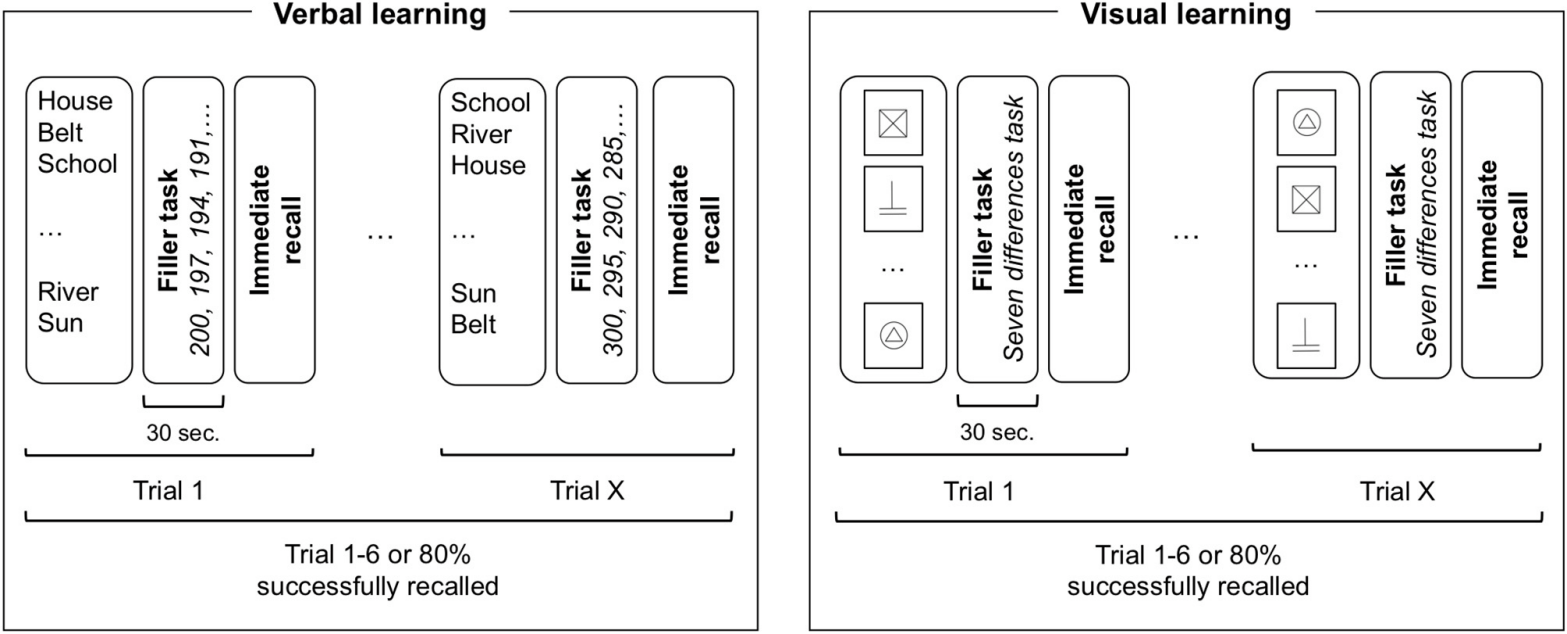
Task design illustration of verbal and visual learning. For each trial, all 15 items are presented in a randomized order followed by a filler task of 30 s before immediate recall. The learning phase is finished when the participant either reaches a learning criterion (80% of items successfully recalled) or after six consecutive trials. Participants performed either verbal or non-verbal task first in a counterbalanced order.

A memory task inspired from Rey's Auditory Verbal Learning Test [RAVLT; (23)], was created including verbal (words) and non-verbal (signs) information. Details from the verbal part have been described in a previous article (21). Stimuli consisted in frequent French words for the verbal part and black and white drawings made out of 1 or 2 basic geometrical forms (circle, square, rectangle, triangle) for the non-verbal part. Task design (illustrated in Figure 1) was similar across modalities. Stimuli were presented by the examiner at a regular pace of 1 per 3 s. After each presentation, a filler task was performed before the free recall. In the verbal part, participants were read 15 words and then asked to count backwards (e.g., 100-1; 100-2) for 30 s as a filler task before recalling the words. In the non-verbal part, participants were presented with 15 black signs on a white card (10 × 10 cm) and asked to look at two similar scenes to find the differences between them during 30 s as a filler task before drawing the signs they learned on a blank piece of paper. To avoid recency or primacy effects, stimuli were presented in a different randomized order for each trial. No recall time limit was imposed.

After a delay of 30 min, participants were asked to freely remember the information. Words were spoken out loud and recorded by the examiner on a form. Drawings were made on a blank piece of paper. A recognition part followed the free recall where participants were asked to recognize the 15 targets among 15 distractors. In the verbal task, distractors consisted in words semantically or phonetically similar to the targets. In the non-verbal task, distractors were similar drawings made out of 1 or 2 basic geometrical forms where objects have been displaced (e.g., mirror image) or exchanged (e.g., circle for square).

#### Scoring Procedure

During learning, the examiner recorded in real-time the number of correct items produced at each learning trial. The end of the learning phase was based on an 80%-success criterion (12 items) or a maximum of six trials to avoid over-learning or discouragement to the task. To account for different number of presentations of the items, a *learning score* was computed by dividing the maximum number of items recalled at any trial with the number of trials necessary to reach the criterion or the end of the learning phase (six trials). A mean total number of produced items was computed by dividing the total of production (correct or incorrect) by the number of trials to complete the learning phase.

In the verbal task, words that did not belong to the 15 targets are qualified as *intrusion errors* and target words said more than one time as *repetition errors*. In the non-verbal task, errors were scored according to an object component (e.g., square instead of triangle) as *object errors* and according to a spatial component (e.g., a circle on top of a square instead of under the square) as *space errors*. Items repeated twice or not recognizable were classified as *other errors*. A mean total error score was computed for the learning phase in each modality, pooling all types of errors.

For retention, *percentage of retention* was computed from the number of correct items freely recalled after 30 min divided by the maximum number of correct items recalled at any learning trial. As for recognition, number of items correctly identified as target or distractors (maximum score = 30) was recorded.

#### Intellectual Functioning

All participants completed the Wechsler scale of intelligence to measure reasoning abilities. Children and adolescents up to 16 years completed the Wechsler Intelligence Scale for Children [WISC-III, IV or V; (24–26)]. From 17 years onwards, the Wechsler Adult Intelligence Scale [WAIS-III or IV; (24, 27)] was administered.

#### Clinical Assessment

In the 22q11DS group, patients and their caregivers were interviewed by a trained psychiatrist using the computerized Diagnostic Interview for Children and Adolescents-Revised [DICA-R; (28)] or the Structured Clinical Interview for DSM-IV Axis I [SCID-I; (29)]. Psychotic disorders and psychotic symptoms were assessed with the supplement of the Schedule for Affective Disorders and Schizophrenia for School-age children Present and Lifetime [K-SADS-PL; (30)] and the Structured Interviewed for Psychosis-Risk Syndromes [SIPS; (31)]. Comorbid psychiatric diagnostics as well as medication are displayed in Table 1.

### Statistical Analyses

Trajectories of performance across trials were examined using mixed model regression analyses in MATLAB R2018b (Mathworks), already reported in previous studies (22, 32, 33). This technique allowed us to examine the trajectory over time of a given variable between two groups and to identify shape differences (i.e., curves that do not follow the same path) or intercept differences (i.e., curves that follow a parallel path but not on the same intercept). Age and IQ were included as covariate variables in the mixed model regression analysis. Between (22q11DS vs. Controls) and within (verbal vs. non-verbal) group comparisons were also conducted in MATLAB with a homemade script. Given the broad age range of the recruited participants, the effect of age was regressed out from the variables of interest before the comparison. For between groups tests we performed Student's *t*-test, Welch's *t*-test (when homoscedasticity assumption is not satisfied) or Mann-Whitney test (when normality assumption is not satisfied). For within groups tests we performed Student's *t*-test or Wilcoxon signed rank test (when normality assumption is not satisfied). Correlation between outcome measures were performed separately for each group (22q11DS and Controls) with non-parametric statistics (Spearman correlation) in SPSS 25 (IBM). When applicable, results were corrected for multiple comparison using the Benjamini-Hochberg method [B-H; (34)].

## Results

### Learning Curves

**Figure 2:**
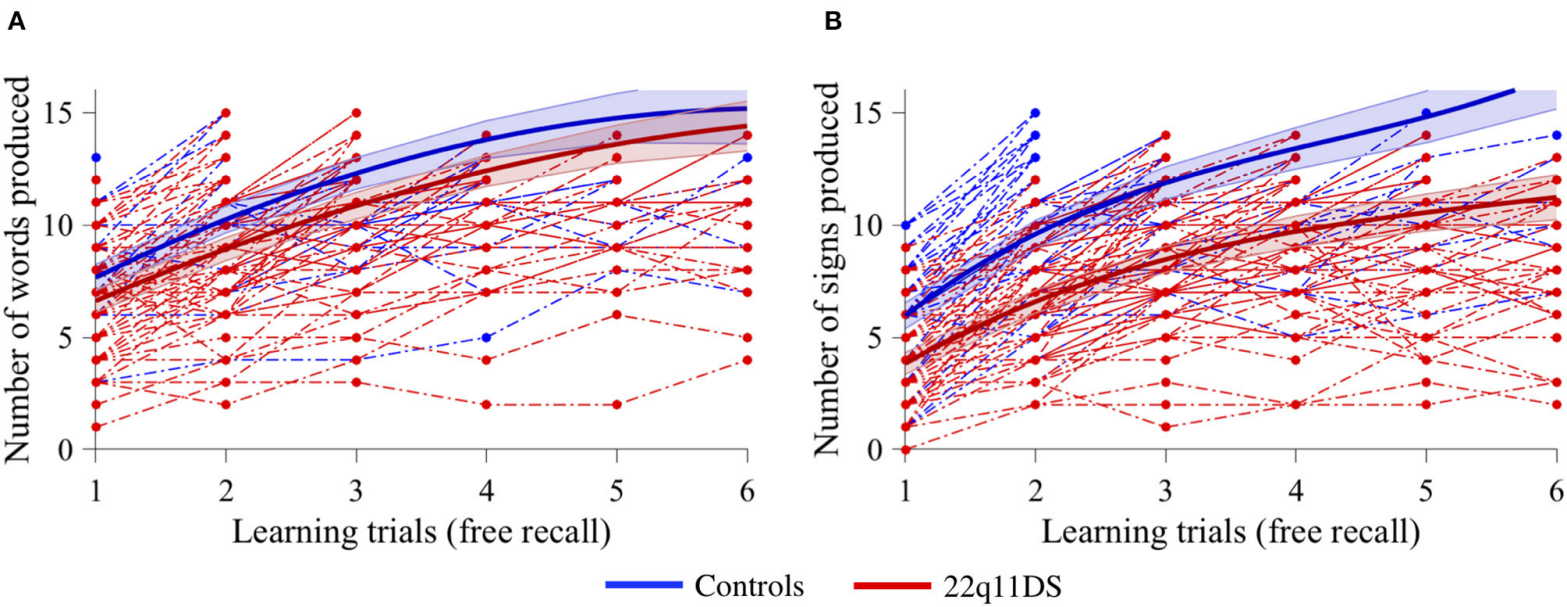
Comparison of learning curves between 22q11DS (displayed in red) and controls (displayed in blue) with age as a covariate for **(A)** verbal information acquisition and **(B)** non-verbal information acquisition.

In the verbal task, a quadratic model best fitted our data. When using age as a covariate, a significant group effect was observed (*p* = 0.010), with lower performance for the 22q11DS group, but no interaction effect with learning trials (*p* = 0.354), indicating that both groups progressed at the same pace trial after trial (Figure 2A). In the non-verbal task, the data also best fitted a quadratic model. When using age as a covariate, there was a significant effect of group (*p* < 0.001), as well as a significant interaction with learning trials (*p* < 0.001). Visual exploration of the data indicated that the 22q11DS group had weaker learning performance from the start and did not improve as fast as the control group during the different trials (Figure 2B).

To account for differences in reasoning abilities, the same previous analyses with full-scale IQ as a covariate were ran and the results remained unchanged. When comparing the amount of trial necessary to reach criterion (80% success), comparable amounts between groups were observed in the verbal task but not in the non-verbal task (Table 2). The 22q11DS group needed significantly more trials to reach criterion.

**Table 2:** Descriptive characteristics and between group comparisons of learning and memory variables.

		**22q11DS**			**Control**			**Independent group comparison**					**Effect size**		**B-H multiple comparison correction**
								**Student's *t*-test**	**Welch's *t*-test**	**Mann-Whitney test**					
**Modality**	**Variable**	**Mean**	**Standard** **Error**	**Median**	**Mean**	**Standard** **Error**	**Median**	***t***	***t***	***U***	***z***	***P***	**Value**	**Range**	**Adjusted *p***
Verbal	Trial to reach criterion	3.82	0.18	3.00	3.30	0.18	3.00	−2.163				0.032	0.38	small	0.012
	Learning score	3.85	0.23	4.00	4.61	0.32	4.00	2.164				0.032	0.38	small	0.010
	Mean total number of items	10.83	0.26	11.00	10.99	0.23	10.67		0.477			0.634	0.08		0.021
	Learning total errors	1.50	0.16	1.33	0.99	0.12	0.79			3076	−2.781	0.005	0.24	small	0.002
	Learning repetition errors	1.19	0.15	0.78	0.78	0.11	0.67			3143	−2.466	0.014	0.22	small	0.004
	Learning intrusion errors	0.31	0.06	0.00	0.21	0.05	0.00			3385	−1.328	0.184	0.12		0.017
	Percentage of retention (30 min.)	86.40	1.87	84.62	85.29	1.78	90.45		−0.461			0.645	0.08		0.023
	Retention total errors	1.35	0.17	1.00	0.75	0.13	0.50			3173	−2.325	0.020	0.20	small	0.008
	Retention repetition errors	1.05	0.16	1.00	0.57	0.11	0.00			3290	−1.775	0.076	0.16		0.015
	Retention intrusion errors	0.30	0.07	0.00	0.18	0.06	0.00			3573	−0.444	0.657	0.04		0.025
	Recognition (30 min.)	29.43	0.12	30.00	29.82	0.08	30.00			4188	2.442	0.015	0.21	small	0.006
	Recognition omission errors	0.43	0.12	0.00	0.09	0.05	0.00			3423	−1.150	0.250	0.10		0.019
	Recognition false recognition errors	0.22	0.06	0.00	0.09	0.06	0.00			3238	−2.019	0.043	0.18	small	0.013
Non-verbal	Trial to reach criterion	4.90	0.15	6.00	3.61	0.20	3.00			2407	−5.408	**<0.001**	0.47	medium	0.003
	Learning score	2.47	0.13	2.17	4.22	0.26	4.00		7.150			**<0.001**	1.33	very large	0.002
	Mean total number of items	10.07	0.22	10.20	10.97	0.22	11.00		3.023			**0.003**	0.51	medium	0.020
	Learning total errors	2.75	0.21	2.33	1.69	0.19	1.38		−3.760			**<0.001**	0.64	medium	0.012
	Learning object errors	1.15	0.09	1.00	0.69	0.08	0.55		−3.968			**<0.001**	0.67	medium	0.008
	Learning space errors	1.14	0.10	1.00	0.82	0.13	0.58	−1.920				0.057	0.34		0.022
	Learning other errors	0.46	0.11	0.20	0.18	0.05	0.00			2877	−3.210	**0.001**	0.28	small	0.017
	Percentage of retention (30 min.)	94.14	1.92	92.86	95.71	1.44	100.00			3703	0.648	0.517	0.06		0.025
	Retention total errors	3.14	0.27	3.00	1.33	0.21	1.00		−5.398			**<0.001**	0.89	large	0.005
	Retention object errors	1.05	0.12	1.00	0.50	0.11	0.00			2819	−3.481	**<0.001**	0.30	medium	0.015
	Retention space errors	1.27	0.16	1.00	0.63	0.14	0.00			2928	−2.972	**0.003**	0.26	small	0.018
	Retention other errors	0.83	0.13	1.00	0.20	0.08	0.00			2742	−3.754	**<0.001**	0.33	medium	0.010
	Recognition (30 min.)	27.81	0.45	29.00	29.26	0.14	29.00			4296	3.583	**<0.001**	0.31	medium	0.013
	Recognition omission errors	0.72	0.16	0.00	0.44	0.11	0.00			3354	−0.862	0.389	0.08		0.023
	Recognition false recognition errors	1.11	0.16	1.00	0.30	0.07	0.00			2670	−4.094	**<0.001**	0.36	medium	0.007

*Significant p-values after Benjamini-Hochberg (B-H) correction are displayed in **bold***.

*Effect size for Student's and Welch's t-tests is reported with Cohen's d and r coefficient for Mann-Whitney test*.

### Learning Score

To account for the number of trials needed, a learning score was computed for each participant on each modality (verbal and non-verbal), as described in the Methods section.

### Data Normality Check

When checking the data for normality, three outliers were identified in the verbal task (one in the 22q11DS group). Since the 5% trimmed mean value was close from the mean value (22q11DS: Mean = 3.882, Trimmed mean = 3.782; Controls: Mean = 4.574, Trimmed mean = 4.358), indicating that those extreme scores do not have a lot of influence on the mean, we chose to include them in the analyses. As security, we ran analyses with and without the outliers and results remained unchanged. Results reported here include the three observed outliers.

#### Between Group Comparison (22q11DS vs. Controls)

As displayed in Table 2, for the verbal learning score, between-group difference was significant but did not survive B-H correction. However, the difference between groups for non-verbal learning score was significant, with more efficient learning in the control group.

#### Within Group Comparison on Learning Score (Verbal vs. Non-verbal)

As shown in Table 3, for 22q11DS, the verbal learning score was significantly higher than the non-verbal learning score. No difference between learning score modality was observed in the control group.

**Table 3:** Descriptive characteristics and within group comparisons of learning and memory variables.

		**Verbal**			**Non-verbal**			**Dependent group comparison**				**Effect size**		**B-H multiple comparison correction**
								**Student's t-test**	**Wilcoxon signed rank test**					
**Group**	**Variable**	**Mean**	**Standard error**	**Median**	**Mean**	**Standard error**	**Median**		***W***	***z***	***P***	**Value**	**Range**	**Adjusted *p***
22q11DS	Learning score	3.85	0.23	4.00	2.47	0.13	2.17	−7.652			**<0.001**	0.90	large	0.008
	Percentage of retention (30 min.)	86.40	1.87	84.62	94.14	1.92	92.86	3.089			**0.003**	0.36	small	0.025
	Recognition (30 min.)	29.43	0.12	30.00	27.81	0.45	29.00		393	−5.17	**<0.001**	0.61	large	0.017
Controls	Learning score	4.61	0.32	4.00	4.22	0.26	4.00	−1.389			0.171	0.19		0.025
	Percentage of retention (30 min.)	85.29	1.78	90.45	95.71	1.44	100.00		1,223	4.49	**<0.001**	0.62	large	0.008
	Recognition (30 min.)	29.82	0.08	30.00	29.26	0.14	29.00		313	−3.56	**<0.001**	0.49	medium	0.017

*Significant p-values after Benjamini-Hochberg (B-H) correction are displayed in **bold***.

*Effect size for Student's t-tests is reported with Cohen's d and r coefficient for Wilcoxon signed rank test*.

#### Spearman Correlation of Learning Scores and Errors During Learning

Both verbal and non-verbal learning scores were significantly correlated amongst each other for 22q11DS and controls (Table 4). Both correlations were of comparable strength (Cohen's *q* = 0.044). No relationship was found between errors committed during learning in the verbal task and the non-verbal task in the 22q11DS group or the control group.

**Table 4:** Spearman correlations for learning and memory variables.

**Group**	**Variable**	***N***	**1**	**2**	**3**	**4**	**5**	**6**	**7**	**8**	**9**	**10**	**11**	**12**
22q11DS	1. Verbal learning score	74	–	−0.103	0.678^**^	0.173	−0.123	0.208	0.580^**^	−0.21	0.301^**^	0.181	−0.162	0.341^**^
	2. Verbal learning total error	74		–	0.444^**^	−0.013	0.466^**^	−0.123	0.023	0.06	0.083	−0.163	0.07	−0.002
	3. Verbal learning mean total number of items	74			–	0.293^*^	0.185	0.153	0.392^**^	−0.074	0.361^**^	0.144	−0.08	0.346^**^
	4. Verbal percentage of retention	74				–	0.065	0.191	0.16	0.039	0.362^**^	0.073	−0.031	0.241^*^
	5. Verbal retention total error	74					–	−0.107	−0.035	0.17	−0.082	−0.283^*^	0.151	−0.008
	6. Verbal recognition	74						–	0.121	−0.021	0.156	0.059	−0.084	0.171
	7. Non-verbal learning score	77							–	0.474^**^	0.349^**^	0.016	−0.399^**^	0.271^*^
	8. Non-verbal learning total error	77								–	0.321^**^	−0.14	0.694^**^	−0.054
	9. Non-verbal learning mean total number of items	77									–	0.037	0.192	0.309^**^
	10. Non-verbal percentage of retention	77										–	−0.344^**^	0.059
	11. Non-verbal retention total error	76											–	−0.046
	12. Non-verbal recognition	77												–
Controls	1. Verbal learning score	53	–	−0.036	0.702^**^	0.548^**^	0.044	−0.086	0.591^**^	−0.068	0.484^**^	0.232	−0.248	−0.044
	2. Verbal learning total error	53		–	0.434^**^	−0.042	0.128	0.01	0.179	−0.165	0.012	0.018	−0.186	−0.255
	3. Verbal learning mean total number of items	53			–	0.478^**^	0.204	0.075	0.517^**^	−0.104	0.422^**^	0.287^*^	−0.300^*^	−0.05
	4. Verbal percentage of retention	53				–	−0.031	0.028	0.403^**^	0.108	0.479^**^	0.098	−0.159	0.068
	5. Verbal retention total error	53					–	−0.197	−0.019	−0.057	0.075	0.227	−0.203	−0.012
	6. Verbal recognition	53						–	−0.067	0.157	−0.023	−0.006	0.111	0.021
	7. Non-verbal learning score	54							–	−0.377^**^	0.525^**^	−0.02	−0.380^**^	0.009
	8. Non-verbal learning total error	54								–	0.343^*^	0.136	0.602^**^	−0.128
	9. Non-verbal learning mean total number of items	54									–	0.24	0.011	0.021
	10. Non-verbal percentage of retention	54										–	−0.072	0.19
	11. Non-verbal retention total error	54											–	−0.071
	12. Non-verbal recognition	54												–

* *p < 0.05*;

** *p < 0.01 (2-tailed)*.

*Significant p-values after Benjamini-Hochberg (B-H) correction are displayed in **bold***.

### Analysis of Errors During Learning Between Groups (22q11DS vs. Controls)

#### Verbal Task

Results from Table 2 show that both groups produced a comparable mean amount of words over the learning phase. The 22q11DS group tended to commit significantly more errors overall, particularly more repetition errors, however these comparisons did not survive the B-H correction. Intrusion errors were not significantly different between groups.

#### Non-verbal Task

Mean signs produced was significantly lower for 22q11DS (see Table 2). Overall they committed more object errors and other errors. Space errors were not significantly different between groups.

### Long-Term Retention (30 min)

Memory retention was compared between groups and modalities using the percentage of information remembered after a delay of 30 min. We found no significant difference between groups in the verbal modality, nor the non-verbal modality (Table 2).

Within groups (see Table 3), the percentage of correctly recalled non-verbal information was significantly higher than the amount of verbal information for 22q11DS and controls.

Spearman correlations of verbal retention and non-verbal retention (Table 4) were not significant in neither group (22q11DS or controls).

### Long-Term Retention Errors Between Groups (22q11DS vs. Controls)

#### Verbal Task

As displayed in Table 2, compared to the controls, the 22q11DS group committed significantly more errors overall, but this comparison did not survive the B-H correction. More specifically, repetition errors were significantly more frequent in the 22q11DS group but did not survive B-H correction. Intrusions errors were not significantly different between groups.

#### Non-verbal Task

As shown in Table 2, significantly higher errors rates were consistently observed in 22q11DS compared to controls. More specifically, a significant difference was observed for object errors and other errors. Conversely, no significant difference was found between groups for space errors.

### Recognition After 30 Min

A significant difference between groups was observed in the verbal and the non-verbal modality (Table 2), however the verbal recognition comparison did not survive B-H correction. Within groups (see Table 3), correctly recognized verbal information was significantly higher (compared to non-verbal) for 22q11DS and controls.

Spearman correlations of verbal and non-verbal recognition (Table 4) were not significant in neither group (22q11DS or controls).

### Recognition Errors After 30 min Between Groups (22q11DS vs. Controls)

#### Verbal Task

Only false recognition errors were significantly higher in the 22q11DS group however, it did not survive the B-H correction (see Table 2).

#### Non-verbal Task

As shown in Table 2, we observed significantly higher rates of false recognition errors in 22q11DS, but no difference in omission errors between groups.

## Discussion

This study aimed to examine dynamics of learning and retention in a large sample of individuals with 22q11DS (compared to controls). By adapting a memory task, groups were compared using parallel task design investigating verbal and non-verbal modalities for learning and retention (with free recall and recognition). In 22q11DS, slower acquisition of non-verbal material was confirmed with higher rates of errors. After a 30 minutes delay, free recall performance (verbal and non-verbal), when corrected for initial learning rate, was similar between 22q11DS and controls. Higher rates of errors for the non-verbal task were observed in patients. Conversely, recognition performance was overall weaker in the 22q11DS group particularly for the non-verbal task and was characterized by specific patterns of errors between modalities.

### Dynamics and Quality of Learning

Investigating dynamics of learning allowed to replicate and confirm the dissociation between verbal and non-verbal information acquisition. More specifically, verbal learning performance between 22q11DS and control groups were not different. Indeed, the patients' group acquired the same amount of information at a similar pace. These results are consistent with previous work describing significantly lower performance in the 22q11DS group compared to healthy controls, although reported performance is in the normal range (about −1 standard deviation to the mean) in tasks of recall of a word-list over multiple trials (4, 11, 12). Conversely, one study reported impaired verbal learning in 22q11DS adults (35). However, almost half of the sample presented with a psychotic illness, and when only participants without any psychotic illness were considered, performance was in the lower normal range (z = −1.30). Altogether, these results confirm preserved verbal rote memory considered a relative strength of the overall cognitive profile of 22q11DS (36). Nevertheless, the specific relationship between psychosis and verbal encoding needs further exploration.

With regards to the non-verbal task, the current study showed similar results as Lepach and Petermann (15). Indeed, a gradual progression of correct answers trial after trial was observed but the total amount of designs correctly recalled at the end of the learning phase was lower compared to controls. It was also smaller than for the verbal task. In a global study on memory investigating non-verbal learning, Lajiness-O'Neill et al. (4) found performance in the lower range on a non-verbal free recall task. Campbell et al. (3) also reported non-verbal immediate memory in the lower range in children and adolescent and Fiksinski et al. (35) in adults. In our sample, a significant difference was highlighted between both groups, with lower performance for 22q11DS compared to controls. Although, the deficit in our non-verbal task seemed of greater intensity compared to previous literature. One possible explanation is that the processes required to complete the task were different across studies. For example, the task from Lajiness-O'Neill et al. (4) required to remember a constellation of dots on a grid, whereas in this study participants had to remember a set of 15 signs presented sequentially.

Analysis of error patterns highlighted generally more errors in both verbal and non-verbal tasks for 22q11DS but the comparison in the verbal task did not survive the B-H correction. Pattern of errors are only rarely reported in previous studies and only two discussed this aspect. In the first study, errors for the non-verbal task were reported but not categorized, which limits interpretations (15). The second study did characterize error type and found significantly more errors of object memory (distortion and size) than spatial memory (misplacement and rotation), in favor of a visual ventral stream deficit hypothesis (13). Similarly, in our study, larger proportions of errors qualified as “object errors” (i.e., square instead of triangle) or “other errors” (not recognizable or duplicates) were observed for 22q11DS patients. As for the verbal task, no specific pattern emerged, with only significantly higher rates of repetition errors for 22q11DS not surviving B-H correction. This could be due to the design of the task regarding the recording of answers. Indeed, words are said aloud with no possibility to verify previous answers (contrary to the non-verbal task where signs are drawn on paper). Remembering which word has been said requires verbal working memory skills, known to be weaker in 22q11DS (17, 37).

### Dynamics and Quality of Retention (Free Recall and Recognition)

Retention was examined in verbal and non-verbal modalities, in two types of settings: free recall and recognition. Both measures were used in a complementary way to fully grasp retention processes.

After 30 min, when corrected for the amount of information acquired in the learning phase, there was no difference in free recall between groups, in both modalities. Indeed, compared to what they have acquired in the learning phase, 22q11DS and controls could freely recall a similar proportion of information. Additionally, for retention percentages, significantly higher percentages of non-verbal information were recalled in 22q11DS and controls. This suggests that even if fewer designs were acquired, they could be retained at comparable rates between groups once memorized. These results imply that, when correcting for the amount of learned information, there is no deficit in the process of retaining the information over a delay of 30 min for 22q11DS patients compared to controls. Calculating the retention percentage according to a measure of learning is a way to shed light on retention without the influence of learning. To our knowledge, no other study except our own (21), has used retention percentages in 22q11DS, so results still need to be replicated in an independent sample. Examining error rates of free recall provided supplementary information on possible mechanisms leading to memory disruption. Patients with 22q11DS committed overall significantly more errors of all types in the non-verbal task, possibly suggesting that the visual information encoded was not very precise or prone to confusions, compared to the verbal task. Results suggest that the trace of visual information is much more labile and tends to change when recalled after a delay in time, whereas verbal information seems more robustly encoded.

With regards to recognition, group comparison showed that 22q11DS had poorer recognition performance in both modalities, but only non-verbal recognition survived the B-H correction. For the verbal modality, results are congruent with Lewandowski et al. (12) who found moderate to large impairment in recognition performance. However, the authors interpreted this result as a weakness of encoding, although scores of encoding were situated in the normal range for that study. Results from Debbané et al. (10) were only partially in line with ours, since the authors did found impaired verbal recognition but reported higher rates of false alarms and commission errors (measures of false recognition) in a verbal task, suggesting source monitoring deficit in 22q11DS. Analysis of error rates in our sample differed significantly between groups, but did not survive B-H correction. This discordance might be a result of the different paradigms used to investigate recognition processes, since the focus of the study from Debbané et al. was specifically on false recognition and omission errors were not recorded or interpreted.

In the non-verbal modality, recognition processes have also been reported as deficient by Lajiness-O'Neill et al. (4). Nevertheless, the comparison should be interpreted with care since both tasks require different processes (face recognition vs. drawing recognition). Analysis of error pattern yielded a significantly higher proportion of false recognition errors in 22q11DS, which could be the result of two mechanisms: increased vulnerability to interference due to deficient executive control (38) and/or a lack of attention to visual details due to inefficient exploration strategies for patients (14).

Overall, results on retention suggest different patterns of impairment depending on type of retrieval (free recall or recognition). Recognition tends to be more challenging for 22q11DS patients compared to controls.

#### Clinical Implications

This work highlights specific patterns of deficits that are useful with regards to educational purposes or the development of tailored intervention in this population. First, by showing similar verbal learning curves between groups, we confirmed previous findings from the literature pointing toward rote verbal memory as a relative strength in 22q11DS. Using the verbal modality to acquire knowledge is a reliable strategy. Secondly, results showed that even if the non-verbal material was more challenging, especially for 22q11DS, the number of presentations of stimuli allowed to increase the number of correctly recalled items. This suggests that repetition is an important tool when working with patients with 22q11DS. Thirdly, analyses of error pattern (learning and retention) indicated that poorer performance for 22q11DS is not related to a lack of answering but from higher rates of errors. Furthermore, higher rates of errors in 22q11DS when asked to recognize targets amongst distractors could suggest that a response option in form of multiple choice is less helpful than for typically developing individuals. Fourthly, finding similar proportion of retained information when correcting for how much was acquired during the learning phase implies that more focus should be on learning conditions and strategies. For example, exploring how the number of items to memorize (smaller portions) or providing efficient encoding strategies (i.e., semantic associations, mental picturing) can improve retention over time would be a next step for the literature. Finally, the positive correlation found between verbal and non-verbal learning scores suggests there could be sub-groups of “poor learners” and “efficient learners” in 22q11DS that need additional help to thrive. In line with this, in many variables Welch's *t*-tests were computed indicating unequal variance between groups and larger variability of scores (larger values of standard deviations) were always found for the 22q11DS group. Again, this highlights the heterogeneity of performance in the 22q11DS group pointing toward sub-groups according to learning skills. Despite a relatively homogenous genetic origin, the heterogeneity of the phenotype of 22q11DS has been extensively documented (39–41). Yet only a handful of studies have focused on the identification of sub-groups of patients based on different variables (20, 42, 43). In a previous study looking at retention of memory over time, we used a cluster analysis to stratify our sample of 22q11DS patients and identify a subgroup of 22q11DS patients with faster memory forgetting rates (21). In the context of this study, identifying “poor learners” and “efficient learners” with a similar technique could allow to conduct more targeted interventions in the 22q11DS population.

### Limitations

First, although stimuli were carefully selected, paying attention to floor or ceiling effects, the non-verbal task was more difficult than the verbal task. This was true for individuals with 22q11DS possibly influenced by specific deficits in visual analyses previously described (14, 44), but also for the typically developing controls. Therefore, it is likely that the abstract nature of the chosen non-verbal stimuli generated an additional difficulty for all participants. Although visual stimuli with semantic connection (i.e., images of objects from daily life) could have been used, we wanted to examine free recall performance, without the influence of verbal processes (i.e., saying out loud the images they remember), which is only possible through abstract drawings. For future work, an alternative solution could be to use more abstract verbal content, for example pseudowords.

Secondly, the task design in both modalities was not exactly similar regarding how the answers were given. Indeed, in the verbal task, answers were spoken aloud to the examiner, whereas in the non-verbal task, participants had to draw on paper. The later response format gives the advantage to review answers that were given. Conversely in the verbal task, participants had to rely on working memory to remember what they had already said not to repeat themselves. This difference might explain why repetition errors were significantly higher in the 22q11DS group, since deficits in verbal working memory have been demonstrated in this syndrome (17, 22, 45).

Finally, 22q11DS is characterized by high rates of psychiatric illnesses (particularly psychosis spectrum disorder, attention deficit disorder) and a high probability to take a medication during the course of their life that could affect cognitive performance (46, 47). In this study we reported comorbid psychiatric illness rates and medication (see Table 1). However, one step further could be to investigates more closely the effects of these potential confounds on the results reported here. For example, stratifying patients based on psychiatric comorbidities or medication could provide additional insight. Nevertheless, the very high comorbidity rates observed in this syndrome with at least half of individuals having two or more psychiatric diagnosis [see (47)] is a major difficulty.

### Conclusion

In conclusion, a memory task was adapted to examine dynamics of learning and retention in both verbal and non-verbal modalities with a parallel design. Consistent with previous research on 22q11DS, learning verbal information was comparable between groups, whereas non-verbal information is acquired more slowly, probably due to inefficient visual processing (especially details' processing). As for retention, analyses demonstrated a different pattern between free recall and recognition. For 22q11DS, when acquired information is considered, there retention rates are comparable controls. Recognition patterns were consistently weaker in 22q11DS, in both modalities. Results from this study are clinically meaningful with regards to educational help and strategies provided for this population. Indeed, as already shown in the literature, verbal modality should be promoted in learning. Furthermore, focus should be on the development of efficient encoding strategies (i.e., semantic associations, mental picturing) that could improve memory.

## Data Availability Statement

The raw data supporting the conclusions of this article will be made available by the authors, without undue reservation, to any qualified researcher.

## Ethics Statement

The studies involving human participants were reviewed and approved by Ethical Committee of the Canton of Geneva (CCER, Switzerland). Written informed consent to participate in this study was provided by the participants' legal guardian/next of kin.

## Author Contributions

JM, MB, MS, and SE conceived and designed the study. JM collected, analyzed the data, and wrote this manuscript. MB and MS provided help in data analysis and interpretation. KB assisted with additional statistical analysis required from the reviewers. MK and SE provided feedback and mentorship on the final manuscript. All authors contributed to the article and approved the submitted version.

## Conflict of Interest

The authors declare that the research was conducted in the absence of any commercial or financial relationships that could be construed as a potential conflict of interest.
